# Can We Six It? Double BASILICA Transcatheter Aortic Valve Replacement in Quadricuspid Aortic Valve

**DOI:** 10.1016/j.jscai.2024.101352

**Published:** 2024-02-29

**Authors:** Nikoloz Shekiladze, Andrew Murphy, Vasilis Babaliaros, Hiroki Ueyama, Adam Greenbaum, Patrick Gleason, Joe Xie

**Affiliations:** Division of Interventional Cardiology, Structural Heart & Valve Center, Department of Medicine, Emory University School of Medicine, Atlanta, Georgia

**Keywords:** aortic valve, congenital heart defect, valve replacement

## Abstract

Coronary artery obstruction is an uncommon yet devastating complication of transcatheter aortic valve replacement (TAVR) and may necessitate leaflet modification. A 38-year-old man presented to our center with quadricuspid aortic valve with severe aortic regurgitation. Double leaflet modification was performed with the Bioprosthetic or native Aortic Scallop Intentional Laceration to prevent Iatrogenic Coronary Artery obstruction (BASILICA) technique prior to TAVR, creating 6 leaflets from 4. The patient tolerated the procedure well with symptomatic improvement. Follow-up transthoracic echocardiogram showed normal bioprosthetic aortic valve function. This case demonstrates feasibility of this procedure with comprehensive preprocedural analysis and intraprocedural imaging guidance.

## History of presentation

**Figure 1:**
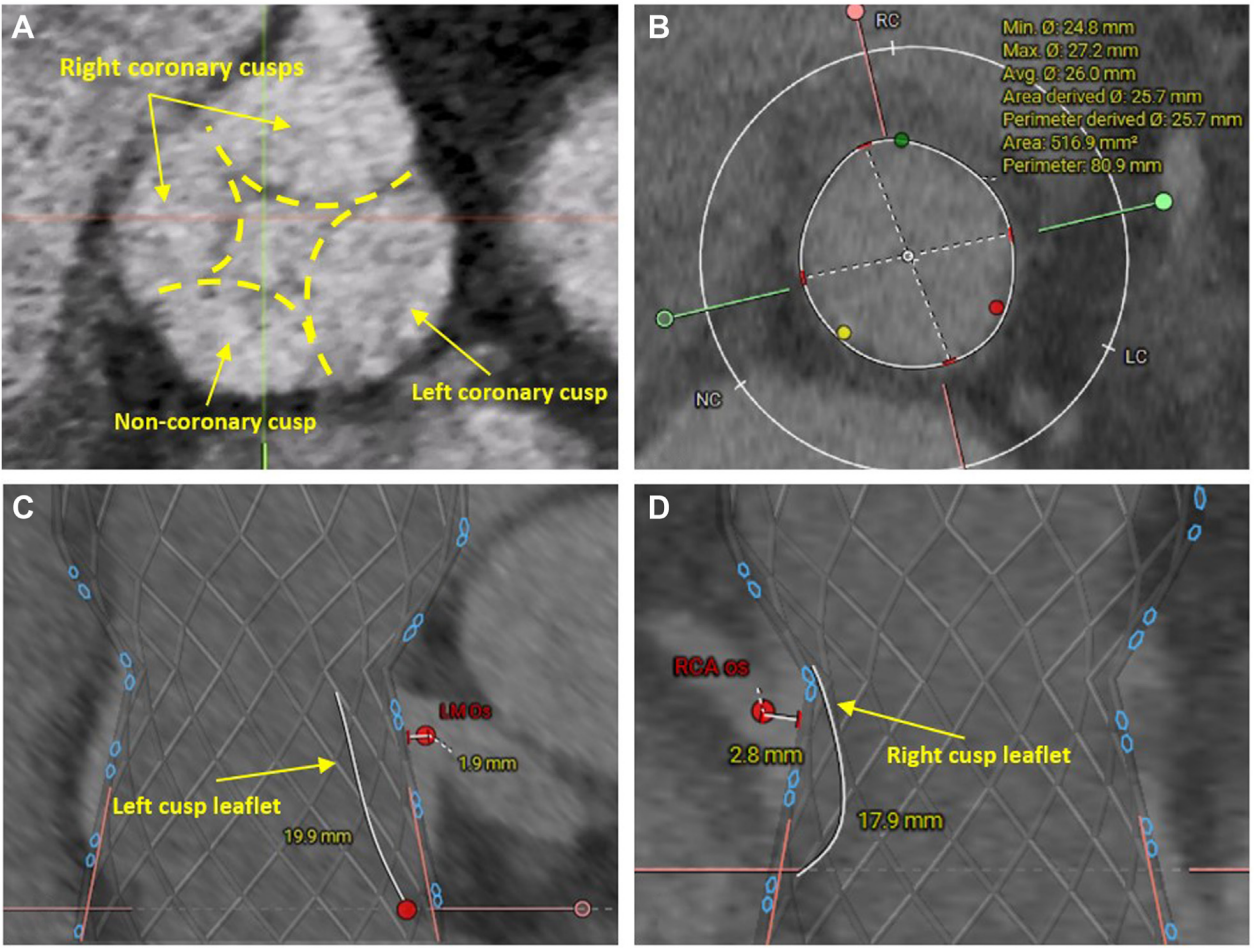
**Computed tomography planning images.** (**A**) Quadricuspid aortic valve morphology seen on cardiac computed tomography images. (**B**) Annular dimensions calculated during mid systole. (**C**,**D**) Predicted deployment of 34-mm Evolut FX (Medtronic) valve raising concern for coronary ostia obstruction and sinus sequestration without leaflet modification due to long leaflets (19.9 and 17.9 mm left and right, respectively) with cusps nearing the sinotubular junction and a low valve to coronary dimension and low valve to sinotubular junction dimension for both the right coronary artery and left main coronary artery.

A 38-year-old man presented to our center for percutaneous management of severe aortic regurgitation (AR) with concomitant moderate aortic stenosis (AS). Medical history was significant for congenital heart disease including a complete atrioventricular septal defect that was repaired in childhood, residual cleft mitral valve with severe mitral regurgitation, a quadricuspid aortic valve (QAV) with severe AR and moderate AS, diabetes mellitus, morbid obesity, nonischemic cardiomyopathy with left ventricular ejection fraction of 45%, and chronic respiratory failure on home oxygen. The patient reported worsening symptoms of dyspnea on minimal exertion, chest pressure, and orthopnea, with an increasing oxygen requirement, and had been admitted several times for decompensated heart failure. Open surgical aortic valve replacement was considered high risk by the congenital heart team owing to his complex medical history, chronic respiratory failure, reduced left ventricular ejection fraction, and obesity. He was referred to our Structural Heart & Valve Center for consideration of percutaneous valve replacement. Evaluation with cardiac computed tomography (CT) confirmed QAV with long leaflets and risk of coronary obstruction following transcatheter aortic valve replacement (TAVR) (Figure 1A, C, D). The procedural plan was double leaflet modification (BASILICA) creating 6 leaflets from 4 and subsequent TAVR using an oversized prosthesis. We opted to address severe AR first as the most downstream valve pathology.

## Investigations

**Figure 2:**
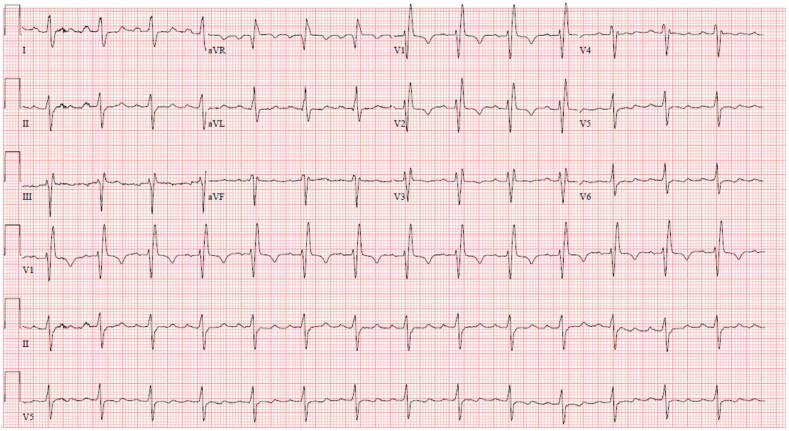
**Baseline electrocardiogram.** Baseline electrocardiogram demonstrating normal sinus rhythm with right bundle branch block and left anterior fascicular block.

**Figure 3:**
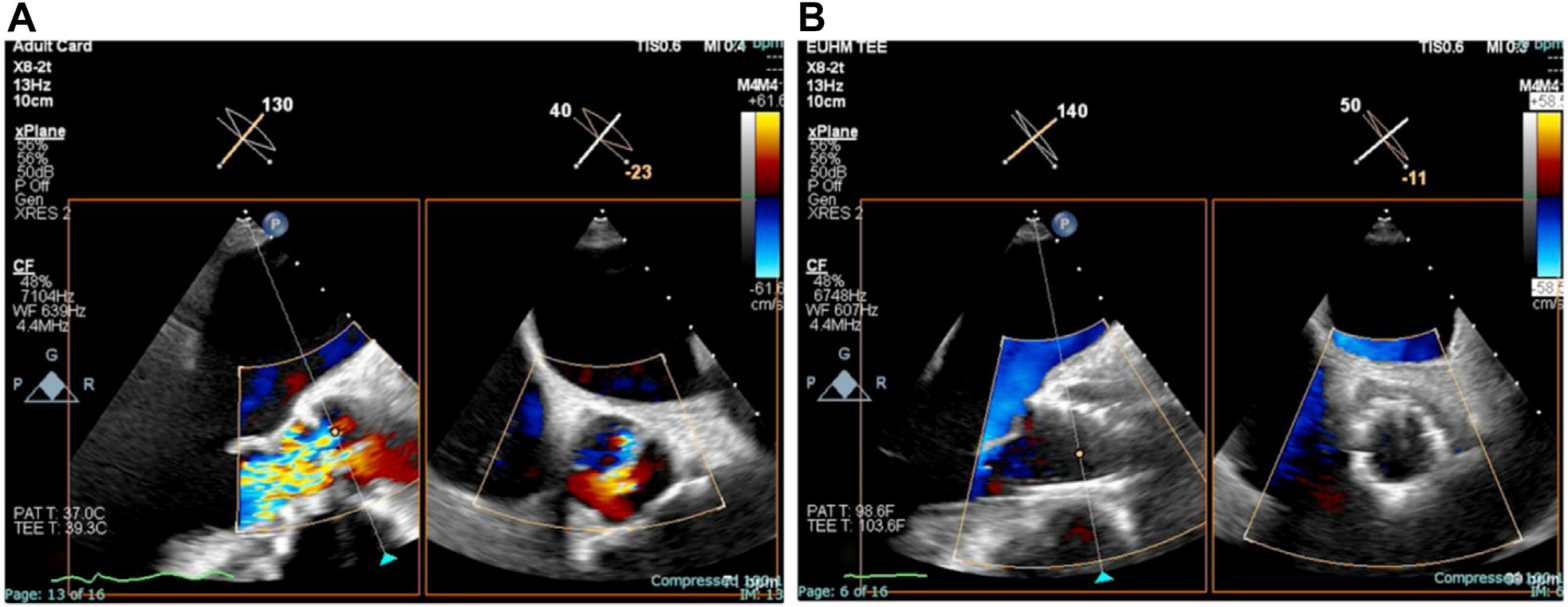
**Transesophageal echocardiography images before and after intervention.** (**A**) Severe aortic insufficiency visualized on short and long axes using transesophageal echocardiogram preprocedurally. (**B**) Postprocedural reduction of aortic valve insufficiency from severe to none.

Pertinent laboratories included severe macrocytic anemia (hemoglobin 7.8 gm/dL), elevated N-terminal prohormone B-type natriuretic peptide (1499 pg/mL). His electrocardiogram demonstrated sinus rhythm with right branch bundle block and left anterior fascicular block (Figure 2), which placed the patient at high risk of complete heart block after TAVR procedure and potential need for permanent pacemaker. Echocardiographic investigation revealed mildly reduced left ventricular function, severe mitral regurgitation, severe AR, and moderate AS (Figure 3A, Supplemental Video 1). Annular dimensions were measured in mid systole using CT, with an area of 516.9 mm^2^, an average diameter of 26 mm, and a perimeter of 81 mm (Figure 1B). Coronary heights were 16.6 mm for the right coronary artery and 11.6 mm for the left main artery. The left and right leaflets were long measuring 19.9 and 17.9 mm, respectively, with the left coronary cusp leaflet almost touching the sinotubular junction (Figure 1C, D). Valve to coronary dimensions were 1.9 and 2.8 mm, respectively, for the left and right coronary ostia. There was no calcification on the leaflets or aortic root.

## Management

TAVR in severe AR associated with congenital aortic valve disease is currently an off-label procedure in United States. In cases of minimal or no annular calcification, the transcatheter valve is intentionally oversized in order to provide adequate seal and anchoring in the aortic annulus. Low valve to coronary and long leaflets placed the patient at increased risk of sinus sequestration and coronary obstruction even with low valve deployment (Figure 1C, D).^1^, ^2^, ^3^, ^4^ Our plan was to perform leaflet modification with double BASILICA and implant a 34-mm Evolut FX valve (Medtronic) given these high-risk features. In our experience during double BASILICA, the decision to cross and lacerate leaflets separately vs crossing both upfront and cutting sequentially depends on several factors, primarily the hemodynamic stability of the patient after crossing and lacerating the first leaflet. We anticipate that crossing both leaflets initially is the correct strategy when feasible. Lacerating sequentially allows the operator to exchange for a pigtail catheter that can be placed in the left ventricle to facilitate prompt TAVR delivery. If the patient becomes hemodynamically unstable, having the other leaflet traversed, snared, and set for laceration helps the operator to move more quickly to valve deployment. Occasionally, in small annular valves, the space in the root could get too crowded and challenging to navigate multiple guides and electrosurgical loops. In that case, we would try to cross and lacerate leaflets separately.

**Figure 4:**
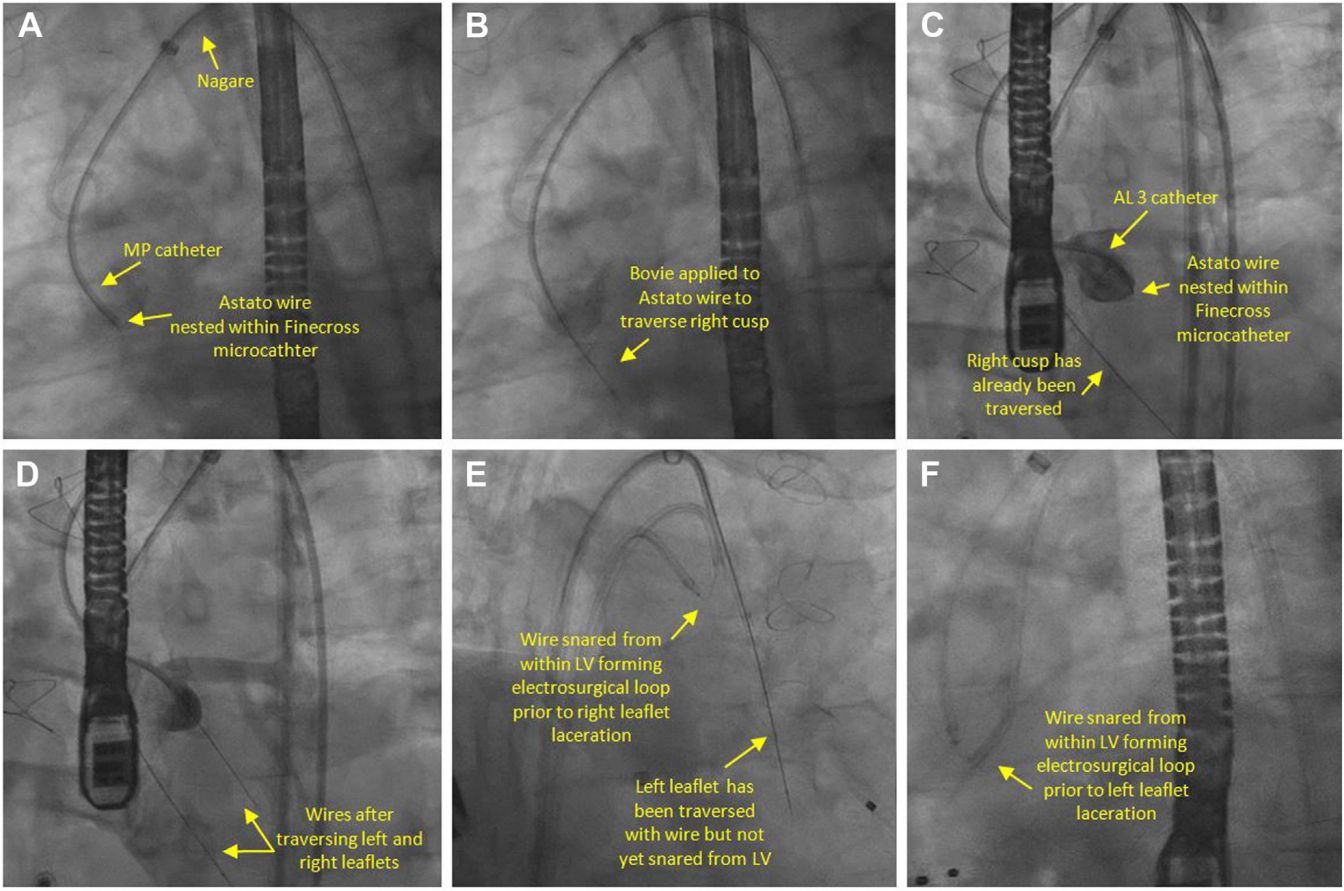
**Aortic valve leaflet traversal and laceration.** (**A-D**) Before and after traversal of right and left aortic valve leaflets, respectively. (**E**, **F**) Laceration of right and left aortic valve leaflets, respectively.

**Figure 5:**
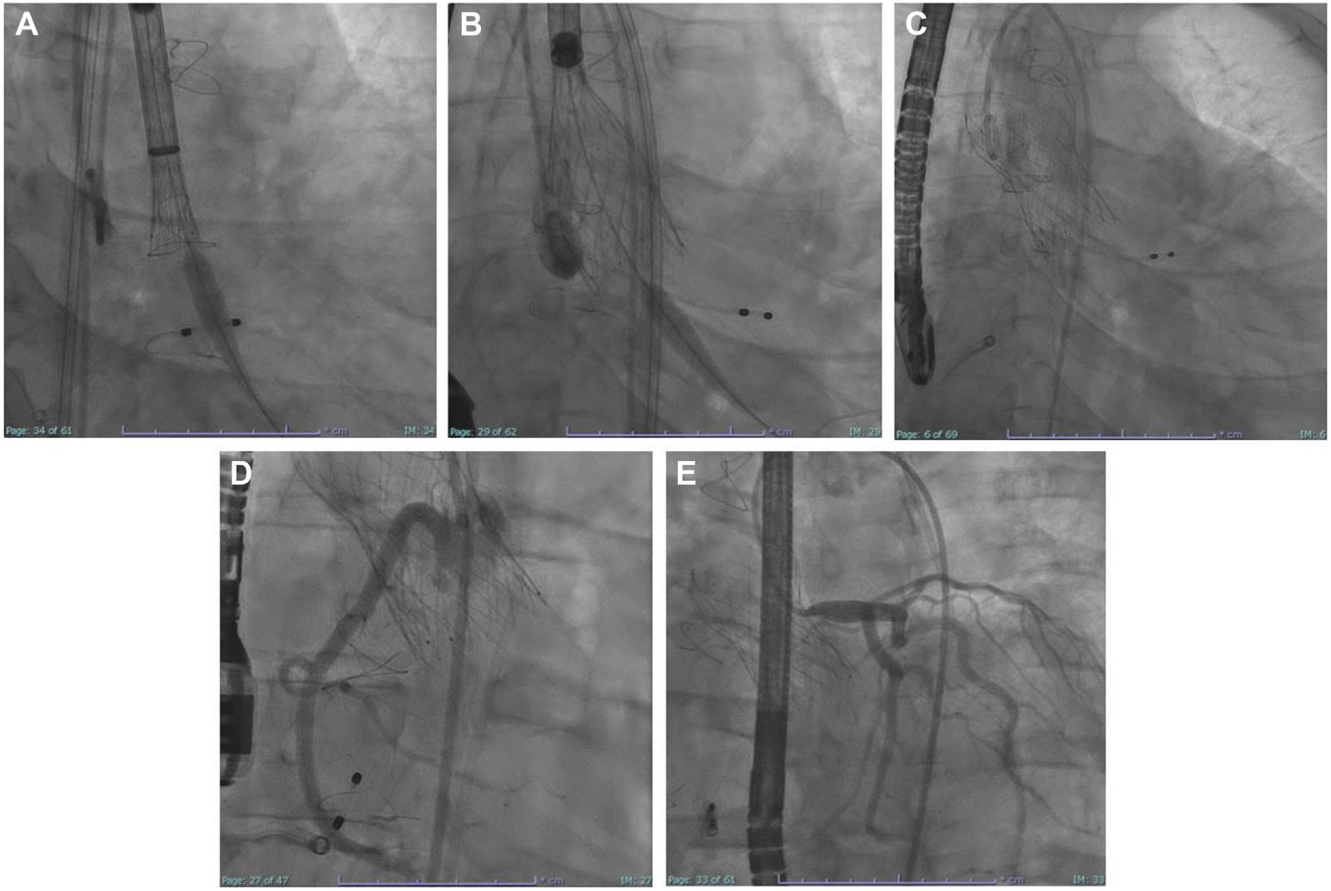
**Aortic valve deployment and angiography.** (**A-C**) Deployment of Evolut FX valve within native aortic valve. (**D, E**) Postdeployment selective angiography demonstrating patency of both coronary ostia.

The procedure was performed under general anesthesia and with transesophageal echocardiogram guidance. Two large-bore 14F DrySeal sheaths (Gore) were placed in the right and left common femoral artery through which the electrosurgical loop was assembled. For the right leaflet, we used 7F multipurpose guide (Medtronic) and for the left 7F Amplatz Left 3 (Medtronic). We were able to successfully traverse at the base of the right and the left leaflets with biplane imaging and predefined CT fluoroscopic angles using an electrified Astato 20 (Asahi Intecc) wire nested within finecross microcatheter (Terumo) (Figure 4A-D, Supplemental Video 2). Bovie (Symmetry Surgical) at 30 W was used on pure cut setting. A Judkins Right 4 (Medtronic) guide and goose neck 30 mm snare (Amplatz Gooseneck; AGA Medical) were used to capture the traversed wire in the left ventricle and externalize it. The Flying V was advanced using a “push-pull” maneuver, and leaflets were then sequentially lacerated (Figure 4E, F, Supplemental Video 2) at 50 W. The patient was able to hemodynamically tolerate leaflet laceration. We then promptly deployed a 34-mm Evolut FS (Medtronic) in standard fashion (Figure 5A-C, Supplemental Video 2) and achieved reduction of AR from severe to none (Figure 3B, Supplemental Video 1), and reduction of invasive aortic transvalvular gradient from 50 to 5 mm Hg. Left ventricular end-diastolic pressure diminished from 35 to 23 mm Hg. Selective coronary angiogram confirmed patency of the right and left coronary ostia following valve implantation (Figure 5D, E, Supplemental Video 2). The patient was successfully extubated postprocedurally and was discharged on the following day. His symptoms have significantly improved. Follow-up transthoracic echocardiogram at 30 days showed normal TAVR gradients and no evidence of residual AR.

## Discussion

Coronary artery obstruction is an uncommon but devastating complication of TAVR, with an occurrence of <1% but with a 30-day mortality nearing 40%.^1^, ^2^, ^3^, ^4^, ^5^ It occurs when a deployed transcatheter heart valve displaces the underlying native or surgical aortic valve leaflets and obstructs the coronary artery ostia. The BASILICA procedure is a transcatheter procedure that entails electrosurgical crossing and laceration of the valve leaflets to prevent coronary obstruction during transcatheter valve implantation.^3^ QAV is reported to have an incidence of approximately 0.013% to 0.043% according to autopsy results.^6^ More than half of patients with QAV developed progressive AR, with AS seen less frequently. Most of these patients with progressive aortic regurgitation require surgery in their fifties or sixties.^7^ There are several published case reports that show TAVR is feasible in QAV with either severe AS or severe AR pathology.^8^^,^^9^ Similar to bicuspid aortic valves, QAVs tend to have long leaflets and are likely to have higher risk for coronary obstruction with TAVR requiring leaflet modification.

## Conclusion

To our knowledge, double BASILICA in QAV has never been performed to date. Our case demonstrates feasibility of the procedure with comprehensive preprocedural analysis and intraprocedural imaging guidance.
